# Transparency and sustainability in global commodity supply chains

**DOI:** 10.1016/j.worlddev.2018.05.025

**Published:** 2019-09

**Authors:** T.A. Gardner, M. Benzie, J. Börner, E. Dawkins, S. Fick, R. Garrett, J. Godar, A. Grimard, S. Lake, R.K. Larsen, N. Mardas, C.L. McDermott, P. Meyfroidt, M. Osbeck, M. Persson, T. Sembres, C. Suavet, B. Strassburg, A. Trevisan, C. West, P. Wolvekamp

**Affiliations:** a Stockholm Environment Institute, Sweden; b Institute for Food and Resource Economics, Center for Development Research, University of Bonn, Germany; c Boston University, United States; d Global Canopy, United Kingdom; e Oxford University, United Kingdom; f Earth and Life Institute, Université catholique de Louvain, Belgium; g F.R.S.–FNRS, Belgium; h Chalmers University, Sweden; i European Forests Institute, Spain; j International Institute for Sustainability, Brazil; k European Commission, Belgium; l University of York, United Kingdom; m BothENDS, Netherlands

**Keywords:** Information, Disclosure, Trade, Deforestation, Agriculture, Forests, Commitments, Soy, Beef, Palm oil

## Abstract

Over the last few decades rapid advances in processes to collect, monitor, disclose, and disseminate information have contributed towards the development of entirely new modes of sustainability governance for global commodity supply chains. However, there has been very little critical appraisal of the contribution made by different transparency initiatives to sustainability and the ways in which they can (and cannot) influence new governance arrangements. Here we seek to strengthen the theoretical underpinning of research and action on supply chain transparency by addressing four questions: (1) What is meant by supply chain transparency? (2) What is the relevance of supply chain transparency to supply chain sustainability governance? (3) What is the current status of supply chain transparency, and what are the strengths and weaknesses of existing initiatives? and (4) What propositions can be advanced for how transparency can have a positive transformative effect on the governance interventions that seek to strengthen sustainability outcomes? We use examples from agricultural supply chains and the zero-deforestation agenda as a focus of our analysis but draw insights that are relevant to the transparency and sustainability of supply chains in general. We propose a typology to distinguish among types of supply chain information that are needed to support improvements in sustainability governance, and illustrate a number of major shortfalls and systematic biases in existing information systems. We also propose a set of ten propositions that, taken together, serve to expose some of the potential pitfalls and undesirable outcomes that may result from (inevitably) limited or poorly designed transparency systems, whilst offering guidance on some of the ways in which greater transparency can make a more effective, lasting and positive contribution to sustainability.

## Highlights

• The links between transparency and sustainabilityare poorly understood.
• We present a typology of information for supply chain governance.
• The coverage of existing transparencyinitiatives is limited and biased in scope.
• We present ten ways in which transparency can improve sustainability governance.

## 1 Introduction

International commodity trade is becoming the mainstay of many of the world’s economies. For example, trade in just a handful of agricultural commodities such as soya, beef, palm oil and timber drives billions of dollars of investment in both producing and consuming nations. The sustainability of how such commodities are produced, traded and consumed is at a critical juncture – with the outcome likely to be decided within the coming decades. Three sets of factors will play a pivotal role in determining whether commodity production and trade can be placed on a more sustainable pathway.

The first relates to the issue of scale. Local consumption patterns of agricultural and other commodities in, for example, North America, Europe, India and China, are increasingly being met by global supply chains rather than local producers (Yu, Feng, & Hubacek, 2013). As a result, the ultimate drivers of environmental and social change in producer countries are often far removed from the places where many impacts materialize. This disconnect between drivers and impacts undermines the ability of local actors in places of both production and consumption to shape their own environments, and emphasizes the need for a commensurately global response (Lambin & Meyfroidt, 2011).

The second set of factors addresses the distribution of responsibility. There is growing recognition of the need for actors involved in every step of global supply chains, and not only producers and consumers, to share the responsibility of placing production systems on a more sustainable footing. Such actors include the traders, processors, retailers and investors that make up global supply chains. They also include state actors as both regulators and major buyers of traded commodities and investors in supply chain infrastructure in their own right, as well as non-state actors who often play pivotal roles in shaping sustainability and rights-based agendas related to international trade. In one high profile example some 190 companies, governments and civil society organizations have signed up to the New York Declaration on Forests that commits signatories to end natural forest loss by 2030, and reduce deforestation by 50% by 2020 (Climate Focus, 2016). Many such commitments have attracted criticism for being tokenistic (e.g. Campbell, 2012, Larsen et al., 2018a), restricted to specific commodities and geographies and lacking any clear implementation strategy. Yet together they represent a concerted decision by both private and public-sector actors to engage in the sustainability agenda. They also give hope to the idea that global value chains can become powerful mechanisms of social and environmental reform (Busch, Oosterveer, Bailey, & Mol, 2015).

The third factor is the explosion in accessible information about how supply chains operate, and the environmental and social risks and opportunities they pose. Labelled by some as the “Information Age” (Esty, 2004), the last few decades have given rise to an era where information transparency processes are increasingly capable of supporting entirely new modes of environmental governance (Fung et al., 2007, Oosterveer and Mol, 2009, Mol, 2009).

The opportunities and risks provided by these new forms of transparency are the focus of this paper. We interpret transparency broadly as a state in which information is made apparent and readily available to certain actors. We also take the view that transparency, in itself, is neither inherently good nor bad, and that the impact of increased transparency depends fundamentally on what information is being made transparent, how, to whom and for what purpose.

On the one hand, increased supply chain transparency can help transform the sustainability of commodity production systems. Transparency can demystify complex supply chains, and help different actors identify and minimize risks and improve conditions on the ground and inform whether and where progress is being made. The inherent complexity of global supply chains has undoubtedly played a central role in masking questionable and unsustainable production practices (Zyglidopoulos & Flemming, 2011). This same lack of transparency has confounded efforts to assess the effectiveness of sustainability commitments made by global actors. Increased public transparency is therefore expected to help rebalance deeply entrenched asymmetries in who has access to information about the origin and impacts of traded commodities, helping to empower vulnerable and concerned actors in both producer and consumer economies (Hall-Matthews and Irby, 2016, Mol, 2010).

Yet on the other hand, supply chain transparency in practice has many shortcomings, from limitations of collecting and disseminating data to the potentially perverse outcomes regarding how the information is used, by whom and to what effect (Mol, 2015, Mol, 2009). Increased transparency is commonly assumed to favour more democratic and emancipatory modes of governance but greater transparency can also exacerbate inequalities and further empower the already powerful (Egels-Zanden et al., 2015, Mol, 2015). Efforts to make highly complex supply chains more transparent will commonly involve a process of simplification, reduction, standardization and dis-embedding from local social and ecological contexts. Such processes will, in turn, make certain attributes more visible while obscuring others. The decision of what information to include and exclude is shaped by dynamics of power (Scott, 1998), with vulnerable actors only empowered if their interests align with those of more powerful actors. At the same time, transparency differs by user and by scale (McDermott, 2014). Information that is transparent, accessible and reproducible to a national government, multi-national corporation or highly resourced international NGO (e.g. registries, scientific studies, databases, detailed written reports) differs from that which is transparent and accessible to local producers and communities.

Despite an explosion of interest and investment in new modes of supply chain sustainability governance (Newton, Agrawal, & Wollenberg, 2013), and a proliferation of supply chain transparency initiatives (Grimard, Lake, Mardas, Godar, & Gardner, 2017), there has been very little critical appraisal of the contribution made by different transparency initiatives and how they can (and cannot) influence new governance arrangements. Moreover, despite the high expectations placed on transparency, there is a lack of clarity as to how improvements in transparency can be designed and implemented to act as a catalyst for positive – and potentially transformative – change.

The aim of this paper is to address these knowledge gaps through four questions that are used to structure the following sections: (1) What is meant by supply chain transparency? (2) What is the relevance of supply chain transparency to supply chain sustainability governance? (3) What is the current status of supply chain transparency, and what are the strengths and weaknesses of existing initiatives? and (4) Based on experiences to date and the current literature, what propositions can be advanced for how transparency can have a positive, transformative effect on the governance interventions that seek to strengthen sustainability outcomes?

In addressing these questions we seek to strengthen the theoretical underpinning of research and action on supply chain transparency, and in particular to advance the notion of transformative transparency as a device to help assess the impacts of new and existing transparency initiatives. We consider transformative transparency as a type of transparency that can help in reshaping human relations with nature and society towards a more sustainable and equitable future, and away from a dominant trajectory of over-consumption, environmental degradation and capital accumulation. Transformative transparency can be further understood as providing decision relevant information, particularly for more vulnerable and disempowered actors, and with an emphasis on information as a means to an end, instead of an end in itself.

We build our analysis primarily on examples from agricultural supply chains and the zero-deforestation agenda, but draw wider lessons for transparency and the sustainability governance of commodity supply chains in general.

## 2 What is meant by supply chain transparency?

The term “transparency” is often used loosely in discourse around sustainability in commodity supply chains. Transparency is often inferred to carry both normative and substantive connotations (Gupta and Mason, 2014, Mol, 2010). Normatively, transparency is often seen as a tool to serve the principles of democracy, participation and accountability. In this sense transparency is viewed by some as having the potential to help overturn deep asymmetries in how different actors access information. Transparency is therefore often interpreted as inherently positive, and of central importance for efforts to create a more emancipatory environmental politics and support bottom-up civil society action (Mol, 2010). In a more substantive sense transparency is typically viewed as encompassing a set of concrete criteria that are necessary to improve sustainability practice and standards, including those related to observation, monitoring, surveillance, mandatory and voluntary disclosure, dissemination, reporting, marketing, complaints and verification.

In the context of corporate accountability, transparency refers to the ability of businesses not only to ‘know internally’ that they are exercising due diligence but also to ‘show externally’ that this is the case (Ruggie, 2011). The latter is particularly important when outsiders, e.g. share- and stakeholders, are concerned about the company’s performance, and more information is required to develop trust and build a positive reputation. As such, transparency can be critical to the credibility of corporate responsibility strategies. Most commodity sectors depend on voluntary (versus regulatory) measures to ensure that such disclosure happens. This underscores the continued lack of transparency regarding many dimensions of commodity production, trade and consumption.

The term “radical transparency” has become increasingly prominent, including in discourse around environmental governance. Heemsbergen (2016) associates radical transparency with third-party disclosure of information that may be involuntarily given off by target actors and used without necessarily having the knowledge or consent of those same target actors. Radical transparency is therefore commonly viewed as being generated by, and dependent on, new digital technologies and data (Zhang, Luna-Reyes, Pardo, & Sayogo, 2016). The changes associated with new levels of transparency are part of a broader wave of technological, social and media change that together make up a rapidly expanding “information scape” that is increasingly embedded and institutionalized in societal structures (e.g. in procurement decisions or third-party accountability frameworks), with potentially transformative effect (Mol, 2015).

Supply chain information has traditionally been disseminated through diverse forms of reporting and disclosure instruments by private and civil society actors, such as company sustainability reports and certification schemes and labels (Egels-Zandén et al., 2015). More recently, many other forms of transparency instruments have proliferated, including online databases, scorecards, self-disclosure information systems, traceability platforms, independent local monitoring initiatives, and various forms of footprint calculators (Grimard et al., 2017).

To unpack the different ways in which the term transparency is used in supply chain research and practice we start by asking: transparency of what? An initial framework of supply chain transparency distinguishes three dimensions of corporate disclosure: (i) the names of the suppliers involved in producing the firm’s products (i.e., traceability), (ii) information about the sustainability conditions associated with these suppliers, and (iii) buying firms’ purchasing and sourcing practices (Egels-Zandén et al., 2015).

Here we further extend this framework to propose a more holistic definition made up of six dimensions of information. This framework of inter-related categories of information describes a recurrent cyclical process of assessment and intervention, which is needed to improve sustainability conditions on the ground. It is comprised of:

1) *Traceability information* that reports on the different actors involved in a supply chain (including production, transport and processing systems), their role, and the nature and rigidity of connections between actors (including contractual and supplier relationships and the power implications thereof) and to production localities. Traceability information provides transparency around associations among actors and between actors and places.
2) *Transaction information* that reports on the purchasing practices and investment decisions of different supply chain actors. This includes commodity purchases, sales of inputs to the commodity production process, and patterns of economic investment and ownership – including by actors outside the primary supply chain. Transaction information helps identify which actors are the main beneficiaries of a given supply chain – and hence who may share responsibility for any sustainability concerns.
3) *Impact information* that reports on social and environmental impacts, as well as other risks associated with specific stages in a supply chain as related to different production, transport, processing and consumption processes. Impact information provides transparency around the sustainability of individual supply chain stages, and thus sets a baseline for assessing the performance of the actors involved.
4) *Policy and commitment information* that refers to the supply chain actors’ policies and commitments to increase the sustainability of their operations, and the processes whereby changes in performance will be assessed (e.g. against current practices or agreed benchmarks*).* Policy information provides transparency on any differences in the levels and strengths of policies adopted by different actors, including sustainability commitments.
5) *Activity information* that reports on actions taken by supply chain actors, e.g. in terms of production, sales, purchasing, processing, and investment decisions, in order to deliver on the targets that are set out by their policies and commitments. Activity information provides transparency on the type and extent of new actions that actors are taking to change their behaviour.
6) *Effectiveness information* that reports on the effectiveness of a given intervention to reduce negative environmental and social impacts and thus improve the performance of a given supply chain actor or production/processing location as set against a specific target, baseline or set of comparators. Effectiveness information provides transparency around how much (or little) progress is being made by a given actor or place.

## 3 The relevance of supply chain transparency to supply chain sustainability governance

Supply chains involve the interplay of actors in three principle sectors: the market, the state and civil society (Lemos and Agrawal, 2006, Newton et al., 2013). The interactions between actors in these sectors has changed markedly in recent years with ongoing globalization processes and the increased prominence of sustainability agendas (Mol, 2015). Two major developments of globalization have played a strong role in shaping efforts to improve the sustainability of commodity supply chains: an increase in the number, reach and complexity of transnational connections, and the relatively limited role of state authorities (Bush, et al., 2015). Both developments, set against a backdrop of rapid change in information technology, have profound implications for how information relevant to the sustainability of supply chains is collected, used and interpreted.

Global supply chains increasingly cross multiple regional and regulatory borders, and the ensuing complexity of material and monetary flows can precipitate myriad unintended effects and telecouplings (Bruckner, Fischer, Tramberend, & Giljum, 2015) including land-use displacement and feedback effects (Lambin and Meyfroidt, 2011, Meyfroidt et al., 2013). In addition, differences in the attributes of specific supply chains and individual companies, together with the increased involvement of non-state actors in shaping supply chain governance arrangements, has dramatically diversified the ways in which sustainability norms are expressed, and how accountability towards such norms is experienced by different actors (Rueda, Garret, & Lambin, 2016). Recent years have witnessed a rapid growth in both private-sector led and hybrid governance arrangements and interventions that rely upon the participation of state, market and civil society actors (Bush et al., 2015, Heilmayr and Lambin, 2016, Lambin et al., 2014). These interventions – whether in the form of new or modified institutions, policy instruments, incentives or changes in access to information – can influence the sustainability of commodity production processes directly, by changing producer behaviour, or indirectly by changing how downstream buyers and investors interact with producers (Newton et al., 2013).

### 3.1 Governance of global supply chains in the information age

Supply chains are embedded in a wider societal context, and are thus influenced by a wide range of social structures and processes (Polanyi, 1944, Uzzi, 1997). The exchange of information amongst actors is one such influence that can play a profoundly important role in determining organizational and economic outcomes in supply chains by shaping actors’ norms, values and decisions (Uzzi, 1997). In recent years the onset of the “Information Age”, driven in particular by technological advancements in the collection, storage, dissemination and interpretation of data, has greatly increased the number of potential opportunities for information exchange.

The growth in open data portals, in particular, has inspired and strengthened new forms of governance intervention. A convincing example of this can be seen in Brazil's open access satellite deforestation monitoring programs, PRODES and DETER, that have revolutionised forest law enforcement in the Amazon by enabling federal police agents and private companies to identify properties that have deforested illegally (Börner et al., 2015, Gibbs et al., 2015, Nepstad et al., 2014).

Information exchange increases connectivity between distant producer and consumer systems by revealing previously hidden “telecouplings” (Liu et al., 2013). Recognition of these telecouplings has increasingly shaped how production systems are governed on the ground through the signalling of information about these systems, e.g. through product labelling or blacklisted properties, which leads to differential sourcing and investment decisions by downstream actors (Garrett et al., 2013, Eakin et al., 2017). Exposing the complexity of these telecouplings underscores the increasing importance of transnational private-sector led and hybrid governance mechanisms that work across and through supply chains, rather than within the management operations of individual actors (Bush et al., 2015, Gereffi and Lee, 2016). The large distances and numbers of actors involved in such arrangements further elevates the importance of information flows between supply chain and other actors, and the need for such information to be comprehensive, credible and transparent.

### 3.2 Information, information transparency, and supply chain governance

**Fig. 1:**
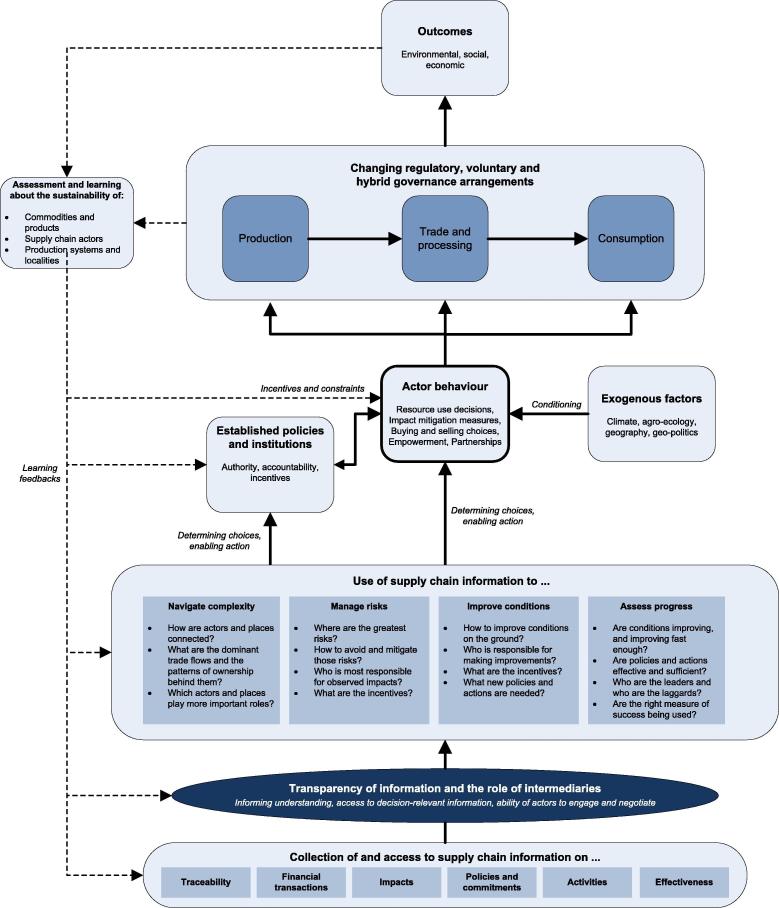
The relationships between supply chain information, transparency, and supply chain sustainability governance. Different levels of transparency mediate how information is used to shape decisions for sustainability governance of supply chains, influence actor behaviour, and determine social and environmental outcomes.

Despite the recent boom in both the modalities of supply chain sustainability governance and the availability of new information regarding the workings, sustainability impacts and performance of supply chains, conceptual understanding of the linkages between supply chain governance and transparency remains weak (Egels-Zanden et al., 2015, Mol, 2015). In this section we outline a simple conceptual framework to help advance this understanding (Fig. 1).

As introduced above we suggest six broad classes of information that can be thought of as relevant to our understanding of the sustainability governance of supply chains; from information used to understand the roles and relationships of different actors, to information on impacts and the effectiveness of policies and actions designed to address those impacts. These different types of supply chain information constitute critical knowledge for decision making and negotiation processes by raising awareness and providing evidence about the impacts of existing behaviours, as well as potential alternatives (e.g. Clark et al., 2011). Yet the way in which supply chain information is taken up and used to improve understanding and decision making is strongly mediated by the extent to which information is transparent to different actors (Fig. 1). Differential access to different types of information limits and sets boundaries on the extent to which different actors are aware of - and able to interpret and use - information, including differences in actors’ awareness of available options (e.g. different regulatory, management and monitoring approaches), and their capacity to assess the risks and benefits that are likely to be associated with each. This underscores the vital enabling role played by intermediary organizations that collect, process and disseminate information on behalf of others (Hess, 2007).

The use of different types of information, as mediated by different transparency processes and access limitations, can help inform and shape decisions relating to the four core challenges of supply chain sustainability governance (Fig. 1): (1) How to untangle the complexity of global supply chains and identify starting points (e.g. specific places or actors) for efforts to improve their sustainability, (2) How to manage the different risks associated with unsustainable production and trade practices, (3) How to improve conditions on the ground, and (4) How to assess progress against different targets and baselines, and understand the extent to which a given set of interventions places the trade of a given commodity on a more sustainable or even transformational path.

Efforts to address these core challenges influence the established policies and institutions that regulate, hold to account and incentivize supply chain actors, as well as the behaviour of different supply chain actors themselves. Changes in actor behaviour – varyingly informed by access to different types and levels of information – including via the options and constraints that shape buying and selling and resource use decisions, as well as the formation of partnerships and changes in status, ultimately shape the emergent supply chain governance arrangements (Fig. 1). This includes the extent to which sustainability outcomes are determined more by regulatory measures, company level Corporate Social Responsibility (CSR) activity, smallholder innovations, downstream pressure from consumer-facing actors and investors who may have made strong sustainability commitments, and/or system wide arrangements such as certification and roundtable schemes and different types of multi-stakeholder initiative (Bush et al., 2015, Rueda et al., 2016). Any such system is of course highly dynamic, with learning feedbacks that can reshape policies and institutional arrangements, as well as the behaviour of individual supply chain actors being motivated by both the changing economic and political situation, as well as changes in how the sustainability of individual traded products, supply chain actors and production localities are measured and perceived (Fig. 1). These feedbacks also influence the ongoing development of informational and transparency processes and institutions, helping, in turn, to either further consolidate existing governance arrangements or precipitate the adoption of new ideas and approaches (Fig. 1).

Supply chain governance arrangements are rapidly evolving in response to such feedbacks. One major change is the imposition of new production standards by powerful downstream actors, on upstream suppliers and producers, such as zero deforestation requirements by retailers, consumer goods companies or investors. The manifestation of such hierarchical modes of global value chain governance (Gereffi et al., 2005) is often mediated by contextual factors and external support, for example through the work of governments in producer regions and civil society actors to help smallholder producers meet stringent sourcing standards. The consequences of this pressure from powerful downstream actors can have rapid and far-reaching consequences for producers, which can be both positive, for example by accelerating access to higher-value and fairer trade markets, and negative, for example by potentially exacerbating existing inequities and precipitating unexpected, negative reactions by both producers and their governments. A clear example of such a negative reaction was the dissolution of the ambitious palm oil IPOP pledge in Indonesia in response to strong government opposition (Seymour & Busch, 2016).

Another major change in how supply chains are governed has been the emergence of a diverse array of new normative and regulatory practices that encompass the interaction between supply chain companies and a wider set of state and non-state actors involved in shaping both production and consumption practices, and leading to a confluence of private, social and public governance (Gereffi and Lee, 2016, Ponte and Sturgeon, 2014). This includes the rapidly increasing array of multi-stakeholder processes, roundtables and hybrid governance arrangements, such as certification schemes. Such arrangements are playing an increasingly prominent and sometimes effective role in fostering supply chain sustainability (Heilmayr and Lambin, 2016, Lambin et al., 2014, Nepstad et al., 2014). A central motivation behind their emergence is the recognition of an urgent need, especially in many developing country contexts, to strengthen the role of the state in order to establish the necessary levels of accountability behind the management of natural resources (McCarthy, 2012, Ponte, 2014).

### 3.3 Impacts of supply chain transparency on environmental and social outcomes

Rapid changes in information access, underpinned by a boom in transparency initiatives are playing an ever more prominent role in shaping evolving supply chain governance arrangements, often with unexpected consequences. On the positive side, access to information on how a given supply chain actor is connected to specific production regions, and the sustainability conditions and challenges associated with those regions, is essential for making informed choices around sustainable sourcing strategies and investment decisions – both to reduce risk in supply chains, and improve conditions on the ground in places where production practices are unsustainable or unfair (Rueda et al., 2016). Such information can serve both consumers and buyers in their ability to make informed choices about the products they buy, and producers and suppliers in their ability to demonstrate that they are adopting improved standards (Egels-Zandén & Hansson, 2015). From the perspective of governing authorities and third-party verification bodies, information on supply chain processes and associated risks is similarly vital for effectively enforcing both regulatory measures such as moratoria, fines, embargos, and taxation, as well as incentive measures such as subsidies and credit lines. Third-parties need the same information to demonstrate the validity of standard systems, target more ethical investments and operate credible complaint procedures and media campaigns. This information is also the basis on which partnerships for sustainability (e.g. roundtables and industry-wide agreements) are built.

Differences in the access to, and use of, new information by different actors means that investments to increase one form of transparency can result in trade-offs between different normative goals and objectives (e.g. Dingwerth and Eichinger, 2010, McDermott, 2014, Mol, 2015). For example, national satellite monitoring systems are transparent and usable by government agencies, companies and well-resourced NGOs, but largely inaccessible to local producers, who often lack secure land and resource rights and the ability to defend their interests against external appropriation (McDermott, 2014). In a similar way, increased demand for greater standardization and harmonization of key pieces of information (e.g. around specific indicators of deforestation and risk at large spatial scales) to ensure comparability (e.g. for the enforcement of standards) can dilute the transparency and accessibility that is afforded to more context-specific environmental and social information that is likely to have greater legitimacy with local actors (Egels-Zandén et al., 2015). Efforts to engage private sector actors in the sustainability agenda can face a strong trade-off in the use of transparency initiatives to name and shame individual companies and governments for non-compliance or unsustainable behaviour (e.g. through sustainability score-cards). Such actions can have a very powerful impact in the short-term but they also risk eroding the trust, confidence and commitments by the same actors that are necessary to drive long-term change. This can result in knee-jerk reactions such as divestment by companies from areas of high deforestation and a de facto embargo of exactly the places that are in greatest need of attention. Finally, and perhaps most importantly, increased attention towards supply chain transparency of any kind can risk blurring the distinction between transparency as a means to an end (i.e. more sustainable production) or as an end in itself. This raises challenging questions about who - and for what - transparency is really for, and whether it is more beneficial to consumers as an indicator of quality in its own right, or producers as a means of helping to improve conditions on the ground (Mol, 2015).

## 4 The current status of transparency in agricultural commodity supply chains

The complexity of global agricultural commodity supply chains, coupled with the relative immaturity of the sustainability agenda in global commodity markets, means that credible and relevant information on supply chain sustainability is often in short supply, presenting a major barrier to effective governance (Boström, Jönsson, Lockie, Mol, & Oosterveer, 2015).

To help take stock of the current status of supply chain information and information transparency – as linked to agricultural commodities in particular – we map some of the most prominent information platforms and initiatives that address one or more of the six main classes of supply chain information: traceability, financial transactions, impact, policies and commitments, activities and effectiveness (Table 1). This review builds on an online survey conducted by the Supply Chain Transparency Network (SCTN) between April and June 2016, with practitioner organizations that manage information platforms and public-access databases related to supply chain transparency (Grimard et al., 2017). The survey reached 24 organizations, including those that had participated in person in at least one of the SCTN meetings in Paris, November 2015 and Oxford April 2016. The survey collected information on the coverage and focus (commodity, geography, time-period, actors) of different initiatives, as well as the data sources, primary audience, limitations and obstacles to effectiveness, outputs and evidence of impact. The review presented in Table 1 supplements the results of the survey with additional initiatives known to the author team, including those that have emerged since the survey was carried out. The review is intended to be illustrative, and we believe it is representative of the information platforms commonly in use by organizations working on supply chain transparency, but it is not exhaustive. We summarize the different types of information system used, typical sources of information, example initiatives, the actors that are primarily involved in producing and using the data, as well as applications and limitations. Whilst it does not provide a complete coverage of all supply chain transparency platforms it is possible to draw some useful conclusions on the state of supply chain transparency from this analysis.

**Table 1:** Information systems in support of sustainability in agricultural commodity supply chains.

Type of supply chain information	Type of information system	Typical information used	Example initiatives (in varying stages of implementation)	Who primarily produces the data?	Who primarily uses the data?	Pathway of influence and intended impacts	Limitations and unintended consequences
Traceability information linking places and actors (Type 1)	Traceability data linking supply-chain actors to production places	Trade data, Bills of Lading, customs data, public and private supply chain logistics data, chain-of-custody certification	Wilmaŕs Open Palm, KnownSources, Trase, Geotraceability, Sourcemap, Provenance	Mostly private providers, some NGOs	Private traders and buyers, investors, consumer groups	Awareness raising, sourcing decisions, risk management, building coalitions of supply chain actors	Information is often confidential and private, limited to specific companies and other actors, coverage often limited to fragments of a supply chain
Financial transactions of supply chain actors (Type 2)	Information on patterns of investment and ownership, values of traded shipments	Sales and taxation data, ownership and subsidiarity information, bond and share investments, loans by banks and pension funds, debt underwriting, commercial bills of lading	Bloomberg, Thomson Reuters, GFW Finance	Private providers, some NGOs	Banks, investors, journalists, campaigners	Sustainable investment strategies, campaigning	Very little information easily accessible in the public domain, especially on sales and purchases
Sustainability impacts and condition information along the supply chain (Type 3)	*Information on sustainability conditions associated with commodity producers*						
	Territorial and jurisdictional mapping and regional scorecards of environmental and social impacts of commodity production, rights and ownership issues	Geospatial observations of sustainability, governance conditions, tenure, using remote sensing and crowdsourcing data	Global Forest Watch, INPE, pastagem.org Kepo Hutan, SPOTT, One Map, OSAS, Produce and Protect, Landscape Accounting Framework, CIFOR Atlas, Environmental Justice Atlas, Land Matrix, Landmark, IAN Risk	Almost exclusively NGOs, some private providers	Diverse range of actors	Hotspots of concern, monitoring change in on-the ground performance	Unconnected to downstream supply chain actors, largely limited to production locations (i.e. excluding storage, processing and transport facilities)
	*Information that links actors to sustainability conditions in places of production*						
	Platforms linking individual downstream supply chain actors to conditions at production sites	Integration of traceability data on supply chain operations with geospatial data	Responsible Timber Exchange, BigChainTool, Starling, Global Forest Watch Commodities, Trase, Terras, PalmTrace, Agrotools	NGOs, private providers	Diverse range of actors	Quality assurance, risk management and due diligence, third party accountability	Commonly depends on user-contributed supply chain information and is therefore limited in scope
	*Information on the environmental impacts of commodity consumption*						
	Footprint calculators, lifecycle analyses	Modelled estimates of total sustainability impacts embedded in commodities and products (Input-Output economic models, LCA analyses)	Resource Trade Database Embodied Environmental Impacts, Carbon Trust, FPN Footprint calculator, WWF Footprint calculator	NGOs, IGOs	Governments, NGOs, companies, journalists	Raising awareness, monitoring of total impacts and efficiencies, assessing policy effectiveness	Invariably based on aggregate sample data and lacking information on the sub-national origin of traded commodities as well as the identity of trading companies; complex assumptions allow for frequent misinterpretation
Policy and commitment, activities and effectiveness information concerning sustainability interventions (Types 4,5,6)	Measurements of actor or territorial performance as set against a specific target, baseline or set of comparators						
	Sustainability scorecards of companies and governments, sector analyses and progress reports	Sustainability commitments and policies, actions, direct monitoring and impact assessments	SPOTT, WWF Palm oil Scorecard, WWF soy scorecard, Greenpeace Palm Oil scorecard, Forest 500, Supply Change, Behind the Brands,	Mostly NGOs, private companies collaborating with NGOs	NGOs, journalists, investors, companies	Voluntary and legal accountability processes, public rankings to reward leaders and shame and expose laggards, company risk management	Often limited to policies rather than specific management activities or direct measures of impact and performance. Limited coverage of commodities
	Self-disclosure sustainability platforms	Voluntary disclosure by private companies	CDP, Global Reporting Initiative, Integrated reporting	Private companies, supported by NGOs	NGOs, journalists, investors, companies	Company risk and performance management, accountability processes	Dependent on voluntary disclosure of accurate and sufficiently specific data by private companies, meaning that impacts can readily be overlooked
	Jurisdictional and property level performance systems, information on governance arrangements	Deforestation measures and other sustainability indicators of production jurisdictions and properties	RSPO jurisdictional certification, Landscape Sustainable Production Standard VCS	NGOs, IGOs	Territorial governments, investors, funders	Rewarding more sustainable jurisdictions with market access, investment and cash	Risks creating embargos of low-performing places that are most in need of investments

Certain kinds of information and information system have developed much more rapidly than others, with geospatial and earth observation data being the most obvious example, spearheaded by major collaborative efforts such as Global Forest Watch and underpinned by an increasing number of ever-cheaper remote sensing products (e.g. Hansen et al., 2013). Scorecards that report on the performance of specific companies have also attracted considerable interest, with a wide range of often competing systems being established by different NGOs in recent years, many of which provide information on the same companies.

However, the current “landscape” of supply chain transparency provided by the overview in Table 1 is characterized by a number of distinct gaps and asymmetries in the available information. These include a particular emphasis given to:

1) Specific commodities and countries, with exports of major forest risk commodities such as soy, beef, palm oil and timber and countries with high levels of deforestation, such as Brazil and Indonesia, dominating the picture. In contrast, there is much less coverage of countries where absolute deforestation levels are relatively low but where deforestation rates are rising rapidly (including many African countries), of major consumption markets such as China and India that are key importers of palm oil, soy, timber and leather, or of rising domestic consumption in many major producer countries;
2) A subset of more visible supply chain actors, including major traders, manufacturers and retailers, with far less coverage of actual producers or consumers or the many other kinds of actors that benefit from international trade in agricultural commodities, including investors, credit providers and agrochemical and seed companies;
3) The sustainability conditions associated with production places, particularly regarding measures of deforestation, with far less information available regarding impacts other than deforestation, or the social and environmental impacts of other steps in the supply chain, including processing facilities and impacts associated with transportation and consumption. Where information on other environmental and social impacts is available there is a widespread lack of information on the methods and data sources used to generate such indicators;
4) The sustainability commitments of individual companies and other actors, with comparatively very little information on their actual activities or effectiveness in delivering on their commitments;
5) Sustainability impacts in general, with comparatively little information available on the level and type of sustainability governance conditions characterizing different stages in the production and movement of traded commodities;
6) Information systems that provide descriptive information on the status and performance of specific regions or actors, with very few initiatives focused on linking information between both actors and places;
7) Systems whose primary role is to support monitoring, surveillance and accountability functions, with far fewer initiatives (mostly limited to the private sector) focused on providing actionable information that can support the decision making of specific actors confronted with particular choices, e.g. for a downstream actor to de-risk their supply chain;
8) Information on volumes handled by companies, with a marked paucity of publicly available data on the financial transactions that drive supply chains, or the monetary benefits accrued to different supply chain actors;
9) Initiatives that are led and funded by civil society organizations or private actors for commercial purposes, with far fewer public transparency platforms on supply chain sustainability run by corporate or state actors; and finally;
10) The needs of actors who are themselves distant from the affected localities (e.g. downstream buyers), with very few initiatives catering directly to the needs of local actors (e.g. communities, farmers) as users of supply chain information.

The most common factor cited by the transparency initiatives as an obstacle limiting the effectiveness of their work was a lack of accessible, comprehensive and comparable data (Grimard et al., 2017), with the majority relying on a combination of publicly available company data, remote sensing information and official government statistics.

## 5 Propositions for transformative transparency

Scholars have long debated the factors that shape knowledge acquisition and learning processes, and the extent to which knowledge systems have the potential to be transformative (Kuhn, 1962). In an early contribution to sustainability science, Cash et al. (2003) suggest that knowledge for sustainability is most likely to be influential if it, and the processes that produced it, is perceived to be *salient* to the problem at hand, sufficiently *credible* in its treatment of evidence, and *legitimate* in the eyes of all stakeholders. Fung et al. (2007) study the effectiveness of targeted transparency policies and conclude that transparency systems are most likely to empower specific actors if the information that is being disclosed is *valuable*, *accessible*, *comprehensive* and *comparable*. In this section we seek to go beyond these broad generalizations and draw on the foregoing discussion, supply chain governance literature and the practical experience of the author team to propose a set of ten propositions that, together, can help guide the development of a more influential – and even transformative – system of transparency for the sustainability governance of supply chains. As in the previous sections we use examples predominantly from agricultural commodity supply chains and the zero-deforestation agenda but consider that these propositions as equally relevant for thinking about the design of transparency systems for supply chains of any description. We break these propositions into three groups, pertaining in turn to the *purpose* of information transparency for supply chain sustainability governance, the *type* of information that needs to be made more accessible and transparent, and the *process* of collecting and disseminating information.

### The purpose of information transparency for supply chain sustainability

#### 1 Transparency is only ever a means, not an end

This proposition seems self-evident but its importance cannot be overstated. Transparency is commonly viewed as a good thing, and companies and governments that freely disclose information about their workings and the environmental and social impacts of their activities are viewed as being worthy of praise. Egels-Zandén, et al. (2015) provide a clear example of this in their study of the Swedish garment company Nudie Jeans Co., which set a target of becoming “the most transparent company in the world”. Such normative connotations associated with transparency risk obscuring – or in some cases supplanting – the importance of the very sustainability and social justice outcomes that increased transparency is intended to help deliver. Support for such concerns can be seen, for example, in the risk that transparency can become a valued attribute in its own right, e.g. in some certification markets (Mol and Oosterveer, 2015, Mutersbaugh and Lyon, 2010), as well as the reputational rewards to be gained from simple information disclosure, whether through platforms such as the Global Reporting Initiative (Dingwerth & Eichinger, 2010) or through improvements in company scorecard rankings (e.g. forest500.org and supplychange.org). This trend is also evident in international negotiations on climate change mitigation, where information disclosure and reporting against nationally-determined targets becomes the only legally-binding element in international conventions, as was the case for the 2015 Paris Agreement. Of course, actors need incentives to be more transparent about their operations. However, care is needed to avoid a mixing of means and ends, and the serious risk that transparency does little more than provide a convenient smokescreen against a failure of companies and governments to act. Or worse still, transparency results in actual harm by serving to exacerbate existing inequalities, e.g. by locking non-compliant yet vulnerable actors out of the market place.

#### 2 Transparency is a double-edged sword

Supply chain transparency is more likely to have a transformative effect if it can work simultaneously to strengthen the hand of actors seeking to implement more sustainable practices (supporting and rewarding the “leaders”) whilst also helping to identify and hold to account those who are not (punishing the “laggards”). Employed in this way, information transparency can catalyse a “race to the top” not only by spotlighting successes and failures, but also by helping to chart a trajectory of continuous, iterative improvements in production, trade and consumption practices. This is a delicate balancing act on multiple levels.

Transparency can facilitate change either by strengthening cooperative action or by strengthening top-down compliance (Egels-Zandén et al., 2015). Transparency as a means of cooperation can work by helping equip actors with the information and tools they need to contribute effectively towards collective action problems, including by helping build coalitions of trusted actors who can reduce costs by working together (Boström et al., 2015). By contrast, transparency as a means of compliance works by way of threats. Threats – and associated repercussions – can come from downstream buyers who shift responsibility for improved practice standards to their upstream suppliers, and may drop those suppliers when they do not comply. Threats can also come from campaigning organizations seeking to expose irresponsible behaviour and malpractice, or through surveillance operations by governments and voluntary accountability initiatives.

The appropriate balance between cooperation and compliance depends on context. However, the level of trust that is vested in a given transparency initiative by different users also critically determines the extent to which this initiative is viewed with cynicism and suspicion by a particular group of users as only having one main purpose – e.g. as a “naming and shaming” mechanism used by campaigners against companies, or as a greenwashing tool used by companies to mask a lack of any real progress from consumers (Dingwerth & Eichinger, 2010). Developing a tool that can work effectively in both ways is challenging, particularly when also trying to ratchet up ambition levels for sustainability outcomes whilst keeping the actors needed to deliver on that ambition firmly on board – a third balancing act. Seymour (2017) captured this challenge well in highlighting the challenge of “simultaneously holding companies accountable for [their] performance in implementing their commitments, while encouraging them to engage in transformational change efforts at the jurisdictional scale” as being “a tiny needle’s eye to be threaded indeed”. A fourth balancing act is cost and complexity. It is invariably both cheaper and simpler to develop a transparency system that is effective at "finger pointing" to strengthen accountability processes than it is to delve into the messier world of providing actionable information and open-access decision support capabilities that are effective in fostering real change by different users on the ground. The latter requires a system which is adaptable to different contexts and stakeholder needs, and a process for engagement that may require a step-wise disclosure of information, both of which are critical for building legitimacy and effectiveness to support improvements in both cooperation and compliance.

#### 3 More transparency may have unintended negative outcomes

Increased transparency can have the effect of exacerbating existing inequalities amongst supply chain actors instead of emancipating the disempowered. More powerful, and sophisticated players typically have greater agency to interpret and use transparency information to their own advantage (Gupta, 2010). Such uses can then have negative outcomes for more vulnerable players, such as smallholder farmers who may be held hostage to sustainability standards demanded by buyers and consumers that may be too expensive or difficult for them to implement (Bacon, 2010). This dynamic can be further exacerbated by the difficulties facing smallholders who do follow sustainable practices yet are unable to provide the necessary information and assurances to buyers. More generally, commodity buyers can decide to divest from a poor and poorly governed region because the region doesn’t meet the sustainability standards necessary for that company to deliver on its commitments. This can easily create a twin-track system where the “good” actors all leave the “bad” places – which, in turn, then go from bad to worse. This kind of feedback loop is the opposite of what needs to happen if transparency is to have a positive impact on a systemic level – i.e. to incentivise sustainability initiatives to target the very places that have the weakest levels of environmental governance (Garrett, Carlson, Rueda, & Noojipady, 2016).

Supply chain transparency will only be effective if the users of the information have both the capability and interest to use it. The quality and reliability of the information, and the way in which information is packaged and presented, has an enormous influence on its uptake and potential for empowerment (Mol, 2009). Disclosed information often lacks adequate methodological information and is presented in such a complex, piecemeal and abstract way as to undermine or completely obfuscate its value. Whether inadvertent or purposeful, such an outcome may be all too convenient for actors that are the focus of any disclosure exercise yet for whatever reason are reluctant to change their behaviour too fast. Related to such problems of data integrity and accessibility is a more extreme type of risk where excessive, complex and otherwise hard to interpret pieces of information are further combined with purposeful disinformation – i.e. half-truths and falsehoods –, misleading information users and fundamentally undermining confidence in available information and confounding attempts to put it to its intended use (Lash 2002).

### The type of information transparency for supply chain sustainability

#### 4 What matters the most are changes on the ground, as linked to supply chains

One of the most important applications of supply chain information and transparency is to understand the effectiveness of different efforts to improve sustainability outcomes, and assess the contribution made by different interventions to deliver the changes that are needed (Lambin et al., 2014). However, existing information systems are woefully inadequate for this task (Climate Focus, 2016, Godar et al., 2016).

Supply chain information systems are varyingly focussed on assessing the sustainability of three types of entity: (i) the agricultural commodities themselves – or the products derived from those commodities, (ii) the actors who produce, trade, market or consume agricultural commodities and (iii) the places where the commodities are produced. Yet ultimately the sustainability of commodity production systems, and downstream interventions (e.g. through changes in procurement strategies or certification systems), can only be assessed by observing changes in conditions on the ground, where those commodities are produced and processed. Herein lies the disconnect. Whilst there is a wealth of spatially explicit environmental and social data for many parts of the world, such data are rarely connected in any systematic way to information either on specific commodities or on the supply chain actors connected to those places. By contrast, the majority of sustainability standards and targets – the things that consumers use to discern the impact of their purchases – are associated either with commodities themselves (e.g. through certification schemes) or the actors that market those commodities (e.g. through individual CSR enterprises and corporate commitments) – and rarely with places where the commodities are produced. This disconnect means that the production of certified products and sourcing strategies of zero-deforestation pledged companies alike can shift to areas with low deforestation, meaning that whilst such products and companies may avoid having any direct negative impact, they also fail to have any positive impact either (Garrett et al., 2016).

Thus, there is a need for mechanisms to systematically link sustainability impacts associated with commodities, actors and places, recognising that the performance of any one element is inextricably linked to the performance of the others. The tracking of such interdependencies needs to be at the heart of any effective monitoring or risk assessment program. Such an approach inherently sets a much higher bar on the interpretation of what should be considered good practice in commodity production and processing. For example, a consignment of timber should ideally be assessed not just on the basis of where it came from, but also on the performance of the sawmills – and thus the other places that the same sawmills also source timber from. Without this extra information there is a risk of suppliers simply bifurcating two or more product lines to serve more and less responsible buyers (e.g. Moura-Costa, Moura-Costa, & Barros, 2016, and see Rausch & Gibbs, 2016 for an example in soy).

#### 5 Supply chain maps need to balance detail and scale

It is commonplace to hear that “more information is better” in discussions about mapping and assessing commodity supply chains. This can be seen in geographic mapping exercises, where there is a seemingly insatiable thirst for higher resolution satellite imagery, and in traceability work, with the belief that to be most useful supply chain maps need to connect individual producers to end consumers and preserve the full identity of traded products along the way (Mol & Oosterveer, 2015). Detailed supply chain mapping information may be essential in some circumstances, and for some stakeholder’s needs – for example in demonstrating legal compliance by individual property owners in order to access specific markets or credit lines, or to monitor the effectiveness of farm-level investments. However, there are also strong arguments for first developing a coarser-grained understanding of supply chain connections and associated risks that can be used to set priorities, develop strategy and identify situations where finer-scale information is most needed.

It is often not logistically possible to deploy fine scale information at the scale and within the timeframe that is needed to deliver on ambitious sustainability targets. For example, beyond the lack of digitalized property-level information systems in many agricultural production regions, land tenure remains unclear and contested in many places. In addition, the scale at which action is most urgently needed is not always or only at the level of individual farms, given both the importance of prioritizing and filtering regions that are most in need of attention, and the fact that deforestation and related sustainability challenges invariably require collective and coordinated action across a wide range of neighbouring actors (i.e. some form of jurisdictional approach, Seymour, 2017). Similarly, effective environmental and social governance can often result in the negative impacts of a given commodity moving elsewhere, and to other markets – and unless monitoring and tracking systems encompass the entire region in which that commodity is produced such leakage effects will be missed (Gibbs et al., 2016).

Godar et al. (2016) make the case for initially adopting a “middle-ground” approach to supply chain mapping that compromises detail in return for extent, and is focused, in the first instance, on mapping the trade of a given commodity from sub-regions of production to countries of import, via export and import companies. This jurisdictional approach to supply-chain mapping provides a birds-eye view of the relationship between downstream actors and places of production, allowing quick discrimination of the actors that are connected, for example, to hotspots of deforestation, and similarly allowing downstream actors to easily assess the overall levels of risk they are exposed to, based on conditions across their sourcing regions. Such an approach also provides a way to prioritize those areas where more detailed mapping is needed (e.g. to the level of specific properties), which can be developed on as needed and in parallel with efforts to improve the sustainability of production practices at jurisdictional scales.

#### 6 Greater transparency of one phenomenon can reduce the relevance of another

It is highly unlikely that increased transparency of one phenomenon will not, in some way, diminish the amount of attention that is given to other, less transparent phenomena; the act of making some things more visible invariably makes other things less visible, and therefore less well understood and ultimately less relevant. Transparency is only ever partial and thus, information platforms and digital technologies do not simply contribute towards greater transparency, but rather they fundamentally alter our perceptions of what is more or less important, and the ways in which we make sense of information (Flyverbom, 2016). This has important practical implications for the role transparency plays in efforts to improve supply chain sustainability as it exposes the side-effects and risks of focussing data collection, monitoring and dissemination efforts on some elements over others. Equally transparency, and trust in the information on which it is based, is not the same for all actors. For example, the ways in which data is processed in a global monitoring system may appear transparent to technical experts but appear opaque and/or be distrusted by others. Likewise, indigenous or small-scale production systems may be completely obscure to outside actors (Scott, 1998).

Such risks can be seen at many different levels. Examples include placing excessive emphasis on: (i) specific indicators, such as deforestation, at the expense of monitoring the loss of other non-forest biomes and other environmental and social impacts; (ii) the role of specific actors, such as traders and major retailers over other, less visible but equally powerful actors such as banks or fertilizer companies; (iii) specific commodities, such as soy, over other commodities such as maize, that is often cropped in rotation with soy; and (iv) specific biomes, regions or countries, such as the Amazon or Brazil over other more threatened biomes such as the Chaco and comparatively poorly studied countries such as Bolivia and Paraguay. Of course, many such decisions reflect choices made in the context of limited resources and specific agendas (e.g. zero deforestation commitments), as well as assumptions as to the most effective theory of change for delivering sustainability outcomes (e.g. a focus on certain traders and consumer goods companies by campaigning organizations). Others are normative judgements in response to issues that are receiving more attention from the media and advocacy organizations. Others still are made in response to limitations of data availability, which can themselves precipitate feedbacks and lock-ins regarding stakeholder engagement – with actors either prioritizing or moving away from more information-rich localities.

Regardless of how they are made such decisions may have unintended consequences. Whilst strong arguments are often made not to over-complicate information platforms, particular care is needed to avoid taking the mantra of “less is more” too far by excessively constraining the types of indicators, insights and perspectives that users are exposed to. Oversimplification can lead to obfuscation if the users lack context and understanding as to how and why certain types of data were produced and selected for disclosure and dissemination. Similarly, it is also important to strike the right balance between harmonization of information systems to aid comparability of data (e.g. by selecting a common subset of indicators) and the need to tailor different systems to different contexts and the needs of different users (Boström et al., 2015).

### The process of improving transparency for supply chain sustainability

#### 7 Transparency should be public but also mediated

Mol (2015) defined four types of transparency in supply chains – management transparency, limited to the exchange of information within or between companies; regulatory transparency, limited to disclosure of information to public authorities; consumer transparency, limited to product information related to claims of sustainable production practices; and public transparency, where direct information on the sustainability of production processes and commodity characteristics are made available to the wider public. Whilst all forms of transparency are needed to drive improvements in sustainability, public transparency has the greatest disruptive and thus transformative potential (Gupta, 2010). However, simply placing information in the public domain is not enough. Systems that make information available but not readily interpretable (e.g. through meta-data, sources, methodologies and clear visualizations and summaries) are likely to only benefit the actors doing the disclosing if the information is not actively scrutinized by third parties. This can be seen in the case of companies that are given credit simply for the fact that they are more transparent (Egels-Zandén & Hansson, 2015) – and are thus vulnerable to criticisms of greenwashing (Dingwerth & Eichinger, 2010). Instead, for public transparency initiatives to be effective they need to managed by trusted intermediary actors, or “infomediaries” (Hess, 2007), that are motivated and capable of ensuring that information is curated, updated and presented in a way that is accessible and understandable to users, and that take care to ensure its credibility, i.e. through transparent metadata and due diligence protocols. These same infomediaries can also play a vital role in identifying and attempting to address information poor environments, where there may be economic or political barriers to access, or a lack of necessary institutional capabilities to organize and standardise data into a usable format (Mol, 2009). However, organizations that play this role are still predominantly non-governmental (e.g. Table 1), and lack long-term business models, underscoring concerns about dependability of datasets that otherwise have a vital role to play in reshaping how supply chains are governed.

#### 8 Data overload can result in decision paralysis

An overabundance of data can undermine rather than strengthen understanding by contributing towards increased confusion and uncertainty regarding the underlying facts of a case (Mol, 2009). Indeed, if not curated and organized in an accessible manner, greater transparency and access to more information can have the counterintuitive and perverse effect of paralyzing decision making due to an excess of choices and competing claims and interpretations of credibility and accountability (Koppell, 2015). Fatigued consumers can find it increasingly difficult to navigate a decision-making environment characterized by a burgeoning number of certification labels, standards, definitions, targets and data sources (Egels-Zandén & Hansson, 2015). Actors are starting to react to this threat through collaborative processes designed to coordinate and harmonize efforts, both in terms of joint implementation of sustainability interventions, e.g. through the Tropical Forest Alliance and in terms of monitoring and assessment protocols, including information on methodological approaches, e.g. through the NYDF Assessment Partners and the newly established Accountability Framework. Rapid increases in data availability can also delay action by raising expectations that new data, with higher resolution or greater accuracy, are about to become available.

#### 9 Transparency is best understood as a process of continuous improvement

The disclosure of supply chain information has the potential to be most transformative when it is part of an ongoing cyclical process of learning and implementation – with information being used iteratively to strengthen both decision making and accountability, e.g. by first calling attention to unsustainable production practices; then supporting supply chain actors in making more sustainable decisions; then ensuring that those same actors are held to account, including through state and non-state frameworks, and are not falling behind in delivering on their commitments; then providing updated information to inform renewed efforts to improve practices; and so on. This iterative process continuously shifts the burden of proof between different actors. For example, in eliciting the potential links between a particular traded commodity and deforestation, consumers can legitimately demand proof that their supplier is not contributing to that deforestation. The burden of proof is shifted, away from the need to demonstrate that a commodity is driving deforestation, and towards individual companies, who each need to demonstrate that they are not part of the problem.

#### 10 Transparency is not a substitute for effective environmental and social governance

Whilst the power of transparency can be highly alluring, greater transparency does not necessarily lead to good governance or provide a substitute for it. To the contrary, there is always a risk that transparency initiatives implicitly or explicitly reify existing arrangements of supply chains and governance regimes in situations where private or public actors lack the capacity or interest to hold back harmful projects, and no degree of transparency is likely to change the sustainability or equitability of outcomes (Larsen et al., 2018b).

In this regard, increased emphasis on private sector actors and voluntary standards as drivers of sustainability agendas should not overshadow the urgent need – especially in many export-oriented developing economies – to strengthen the role of the state in overseeing the governance of natural resources and protecting the rights of more vulnerable actors (e.g. McCarthy, 2012, Larsen et al., 2014, Ponte, 2014). Indeed, the emergence of hybrid governance arrangements, where civil society actors often play vital roles in facilitating and mediating the interplay between private and public sector actors, is partly a response to growing concerns about the risks of company overreach when set against weak government institutions and regulations, including regarding land-rights and deforestation (Amengual, 2010, Lambin et al., 2014). As part of this process, voluntary and/or third-party transparency has taken on an even greater significance, substituting in many instances for failures to enforce existing domestic legislation or translate international obligations, including human rights and environmental protections, into binding legislation. Given current governance shortcomings and failures, public transparency initiatives thus have a vital role to play. However, they should be continuously appraised for their ability to support governments and civil society groups in their regulatory and empowerment functions, whilst also guarding against the risk that they may further entrench existing power disparities between multi-national companies and local actors.

## 6 Conclusions

There is no question that one of the defining characteristics of modern society has been an explosion in the way that information is generated, accessed, shared and used to shape individual behaviours and decision making at every level. The field of sustainability and commodity supply chains is no exception. The globalization of trade in agricultural and other commodities has created new connections and interdependencies between distant actors – including consumers, companies and investors – and land-uses that did not exist only a few decades ago (Lambin et al., 2014). Some of these connections carry the risk of exacerbating environmental impacts and existing social inequalities, whilst others offer the potential for a more constructive engagement between private companies, governments and civil society to promote sustainability. Yet, regardless of the outcome, the increased complexity of global commodity supply chains, and the number of actors involved in shaping how they are governed, make the path towards sustainability a more difficult one to follow. They also underscore the increasingly pivotal role of information and information access in delivering sustainability outcomes.

In providing one of the first comprehensive reviews of the interplay between supply chain transparency and supply chain sustainability governance, this paper strengthens the theoretical underpinnings of research and action on supply chain transparency. In doing so it also exposes some of the potential pitfalls and undesirable outcomes that may result from (inevitably) limited or poorly designed transparency systems, whilst offering guidance on some of the ways in which greater transparency can make a more effective, lasting and positive contribution to sustainability.

We present a new typology of supply chain information that is relevant to our understanding of the sustainability governance of supply chains – namely information on supply chain traceability, financial transactions amongst supply chain actors, sustainability impacts, the policies and actions intended to address those impacts, and the effectiveness of any such efforts to improve conditions on the ground. Breaking down the meaning of transparency helps reduce ambiguities and confusion in how information is collected and used to support change processes, whilst also helping to expose biases and imbalances in the kinds of information that is accessible to different actors.

Taken together, these different classes of supply chain information can help actors navigate the complexity of global supply chains, identify and assess options to mitigate and reverse the impacts of unsustainable practices, and monitor and report on progress against long-term goals. Differences in the availability of different kinds of information, and the access that different actors have to the same information, can have a profound influence on the way in which decisions over resource use and commodity trade are made, whilst also shaping who is most likely to win and lose from such decisions. Transparency can be at the same time empowering and disempowering – capable of either fostering cooperation or enforcing compliance – depending on the reasons why and processes by which information is being disclosed and the agency and motivations of the actors involved.

The limited role of state actors in the governance of many supply chains, coupled with the increased dependence on voluntary standards and commitments by private companies, underscores the increasingly prominent role played by intermediaries, often from civil society, to make key datasets publicly available and accessible to a wide range of actors. Designed effectively, public transparency systems can help bolster enfeebled state agencies and support the establishment of independent accountability processes, whilst also helping to provide actionable information for private companies that are committed to making more sustainable choices. Public transparency is also often a foundation stone for successful hybrid governance arrangements that can arise in situations where both state regulations and voluntary measures are found to be wanting.

In taking stock of existing transparency initiatives, we uncovered a number of major shortfalls and systematic biases in the coverage of different kinds of supply chain information. These biases relate not only to the number of commodities and countries that are being mapped and assessed, but also the types of actors and indicators that are assessed, as well as the way in which the data are analysed, presented and explained. Major shortfalls include a comparative absence of information on both the most vulnerable (e.g. smallholders) and the most powerful (e.g. investors) supply chain actors; information on the vertical and horizontal distribution of the economic benefits of commodity production and trade; and information on the extent and effectiveness of any activities that are being implemented to improve sustainability outcomes on the ground.

The path towards a positive, transformative transparency is strewn with obstacles. We present a set of ten propositions that, if taken together, can help guide progress – regarding the purpose of information transparency for sustainability governance, the type of information that needs to be made more accessible and transparent, and the process of collecting and disseminating information. These propositions strengthen the theoretical underpinnings of how enhanced transparency can improve supply chain governance and lead to improved sustainability outcomes. Whilst there are no silver bullets, transparency initiatives are more likely to make a positive contribution if: they go beyond a narrow focus on products and companies to also assess changes on the ground; resist normative interpretations of how transparency should be interpreted or assumptions that more, finer-scale information is always desirable; facilitate greater cooperation amongst actors with shared goals whilst at the same time strengthening compliance where progress is lacking; are hosted primarily in the public domain yet mediated by actors who can help ensure that any information is accessible to those who need it the most; and are firmly anchored within hybrid and state-led governance structures that are concerned first and foremost with positive sustainability and social outcomes and view transparency not as some static commodity but as a dynamic and constantly evolving contribution towards a process of continuous improvement.

Central to the contribution of transparency initiatives to improved sustainability outcomes is trust. Trust is key to achieving the balancing act between greater cooperation and greater compliance and accountability – all of which are needed to drive change (Egels-Zandén et al., 2015). While a degree of transparency can build trust, the use of transparency to replace trust can alter social relations by favouring impersonal means of social control, emphasizing standardized information, external surveillance and third-party auditing. If transparency initiatives are not managed very carefully this social dis-embedding process can undermine voluntary cooperation, the development of shared meaning, and reciprocal relationships of trust and trustworthiness and lead instead to a ‘downward spiral of distrust and control’ (Shapiro, 1987).

Trust and cooperation are also critical factors in choosing the information to be shared by a transparency system. Because all information systems inevitably involve a certain level of simplification and abstraction – which will not be equally interpretable to all actors at all times – their success depends, in part, on a willingness by the same actors to accept the situation and trust the information that is provided, and view it as appropriate for the purpose of informing the decisions at hand (McDermott, 2012). Cooperation between and participation by stakeholders in processes used to prioritize and select information are central to building such trust.

Informational governance is still very much in the making (Mol, 2009, Mol, 2015) and ongoing developments in information technology, data capture and storage, citizen engagement, social media and awareness, will continue to shape the significance and contribution of transparency initiatives – and the implications for different stakeholders – for years to come. The ways in which greater transparency will shape sustainability and social outcomes will ultimately be determined by the response of market actors to the emergence of new and often hybrid governance arrangements involving both state and private sector actors (Gereffi and Lee, 2016, Ponte and Sturgeon, 2014), and the capacities of different actors to contribute towards, use and assimilate the information they need to foster positive changes.

The research community has a major role to play in supporting the process of developing a positive, transformative transparency for supply chain sustainability governance. Open questions remain regarding the ways in which improvements in the availability and accessibility of information can drive improvements in supply chain governance (Mol, 2010). Addressing this requires a better understanding of the biases and shortcomings in the information and information systems that are currently in play; improved methodologies for establishing causal relationships between transparency, governance interventions and improvements in sustainability; and, at a more fundamental level an improved comprehension of the theories of change that underpin informational governance and the role that information plays in shaping cooperation and compliance behaviours between actors in different sectors and at different levels in global supply chains.

## 7 Conflict of interest

We declare that we are not aware of any conflict of interest, including financial, personal or other relationships with other people or organizations within three years of beginning the submitted work that could inappropriately influence, or be perceived to influence, our work.
